# TRP, TRPL and Cacophony Channels Mediate Ca^2+^ Influx and Exocytosis in Photoreceptors Axons in *Drosophila*

**DOI:** 10.1371/journal.pone.0044182

**Published:** 2012-08-31

**Authors:** Guadalupe Astorga, Steffen Härtel, Magdalena Sanhueza, Juan Bacigalupo

**Affiliations:** 1 Department of Biology, Faculty of Sciences, University of Chile, Santiago, Chile; 2 Millennium Institute for Cell Dynamics and Biotechnology, Faculty of Sciences, University of Chile, Santiago, Chile; 3 Laboratory for Scientific Image Analysis, (SCIAN-Lab), Biomedical Neuroscience Institute (BNI), ICBM, Program of Anatomy and Developmental Biology, Faculty of Medicine, University of Chile, Santiago, Chile; Indiana University School of Medicine, United States of America

## Abstract

In *Drosophila* photoreceptors Ca^2+^-permeable channels TRP and TRPL are the targets of phototransduction, occurring in photosensitive microvilli and mediated by a phospholipase C (PLC) pathway. Using a novel *Drosophila* brain slice preparation, we studied the distribution and physiological properties of TRP and TRPL in the lamina of the visual system. Immunohistochemical images revealed considerable expression in photoreceptors axons at the lamina. Other phototransduction proteins are also present, mainly PLC and protein kinase C, while rhodopsin is absent. The voltage-dependent Ca^2+^ channel cacophony is also present there. Measurements in the lamina with the Ca^2+^ fluorescent protein G-CaMP ectopically expressed in photoreceptors, revealed depolarization-induced Ca^2+^ increments mediated by cacophony. Additional Ca^2+^ influx depends on TRP and TRPL, apparently functioning as store-operated channels. Single synaptic boutons resolved in the lamina by FM4-64 fluorescence revealed that vesicle exocytosis depends on cacophony, TRP and TRPL. In the PLC mutant *norpA* bouton labeling was also impaired, implicating an additional modulation by this enzyme. Internal Ca^2+^ also contributes to exocytosis, since this process was reduced after Ca^2+^-store depletion. Therefore, several Ca^2+^ pathways participate in photoreceptor neurotransmitter release: one is activated by depolarization and involves cacophony; this is complemented by internal Ca^2+^ release and the activation of TRP and TRPL coupled to Ca^2+^ depletion of internal reservoirs. PLC may regulate the last two processes. TRP and TRPL would participate in two different functions in distant cellular regions, where they are opened by different mechanisms. This work sheds new light on the mechanism of neurotransmitter release in tonic synapses of non-spiking neurons.

## Introduction

**Figure 1:**
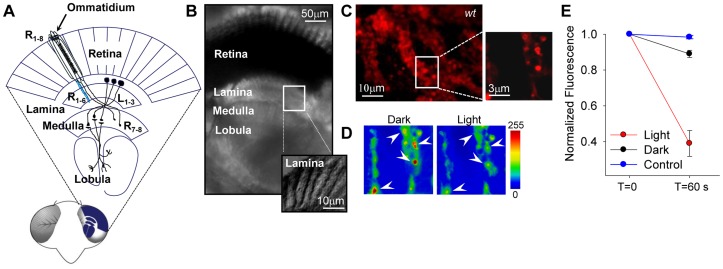
*Drosophila* visual system and brain slices. (**A**) Schematic representation of a section of the fly’s visual system. Photoreceptors somata are arranged in ommatidia in groups of eight (R1–R8). R1–R6 project to the lamina forming a columnar assembly (cartridge) with the axons of the large monopolar neurons (L1–3). (**B**) Microphotograph of a slice preparation of the visual system. (**C**) Synaptic boutons fluorescently labeled with FM4-64 in the lamina of a *wt* fly (see Materials and Methods). The inset shows a detail of the boutons shown in pseudocolor in C. (**D**) Confocal images displaying fluorescence previous (left) and 60 seconds after light (20 s, white light). (**E**) Plot of the normalized mean fluorescence measured in the boutons shown by the arrowheads in D (red circles) 60 seconds (t = 60s) after light exposure (t = 0). The control for FM-464 photobleaching (blue circles) was measured in the abdomen, representing a light insensitive region. Normalized mean fluorescence of boutons from slices not exposed to light is also included (black circles). n = 4. Pseudocolor scale in arbitrary units.

Light transduction in *Drosophila* occurs in retinal microvillar arrangements running along the photoreceptor soma, termed rhabdomere. The axon of this non-spiking neuron releases histamine in a tonic manner [1], [2]. It presents a T-bar ribbon synapse, a particular structure of the active zones specialized for fast and sustained multivesicular neurotransmitter release in response to graded membrane depolarizations. R1–R6 photoreceptors make multiple axo-axonic synaptic contacts with large monopolar (LI-L3) and amacrine cells in the lamina (Fig. 1A). Cell somata are located in the outermost part of this neuropile, leading to a particular situation where axonal arrays (named cartridges) are the predominant components of the lamina. The axons of centrifugal medullar neurons (C2–C3), a T-shaped centripetal neuron (T1) and a wide field tangential neuron (Tan) are also found in the lamina [3], [4]. In the rhabdomere, photon absorption triggers rhodopsin isomerization into an active state which, upon interaction with a G_q_-protein, activates phospholipase C (PLC_β4_). This enzyme, encoded by *norpA* [5], hydrolyses phosphatidylinositol biphosphate (PIP_2_) into inositol trisphosphate (IP_3_) and diacylglycerol (DAG). This signaling cascade has been widely implicated in the activation of TRP and TRPL [6], the two channels carrying the phototransduction current [7], [8]. Although the mechanism of channel gating remains undetermined, there is evidence that under experimental conditions, DAG, polyunsaturated fatty acids (PUFAs) [9], [10], PIP_2_ [11], [12] and protons [13] are involved in opening TRP and TRPL, whereas IP_3_ receptor does not [14], [15]. Interestingly, TRP and TRPL expressed in heterologous systems [16] are activated by Ca^2+^ depletion of the endoplasmic reticulum (ER). Here we confirmed the presence of TRP in the lamina [17], where we report that TRPL is also expressed. For the first time, we provide evidence that these channels are implicated in neurotransmitter release in the lamina, where they apparently allow Ca^2+^ influx via a store-operated channel (SOC) mechanism [18] and could also be regulated by a PLC-mediated cascade [6]. Furthermore, we show that the voltage-dependent Ca^2+^ channel cacophony, the only fly homologue of vertebrate N-, P/Q- and R-type [17], [19], [20], is present in the lamina where it plays an important role in photoreceptor synaptic transmission, probably as a first step in a complex cascade involving both intracellular and extracellular Ca^2+^ signalling.

These results contribute to understand tonic neurotransmitter release in ribbon-type synapses and presynaptic enhancement by intracellular Ca^2+^ in non-spiking neurons.

## Results

### TRP, TRPL, Other Phototransduction Proteins and Cacophony are Present in Photoreceptors Axons

**Figure 2:**
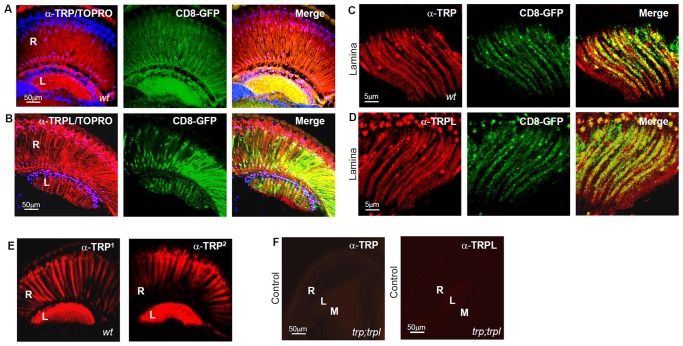
TRP and TRPL channels in *Drosophila* visual system. Confocal immunofluorescence images of TRP and TRPL in *wt* brain slices. (**A**) Single confocal optical sections showing TRP distribution (red) in the retina (R) and lamina (L) detected with a monoclonal α-TRP antibody. Nuclei stained with TOPRO (blue) (left). Photoreceptors labeled green by ectopic expression of CD8::GFP (center). Merge (right). (**B**) Same as in A, for TRPL distribution detected by monoclonal α-TRPL antibody reactivity. (**C**) Z-projections of 10 confocal optical sections (Δz = 0.3 mm) presenting a higher magnification view of TRP immunostaining in the lamina of *wt* brain slices. (**D**) Same as in C, **s**howing TRPL immunostaining. (**E**) TRP immunolabeling in the retina and lamina with two different monoclonal antibodies, α-TRP^1^ and α-TRP^2^. (**F**) Negative controls for α-TRP (top) and α-TRPL (bottom) antibodies, tested on a *trpl^302^;trp^CM^* null double mutant. Same laser settings as for the images in A.

**Figure 3:**
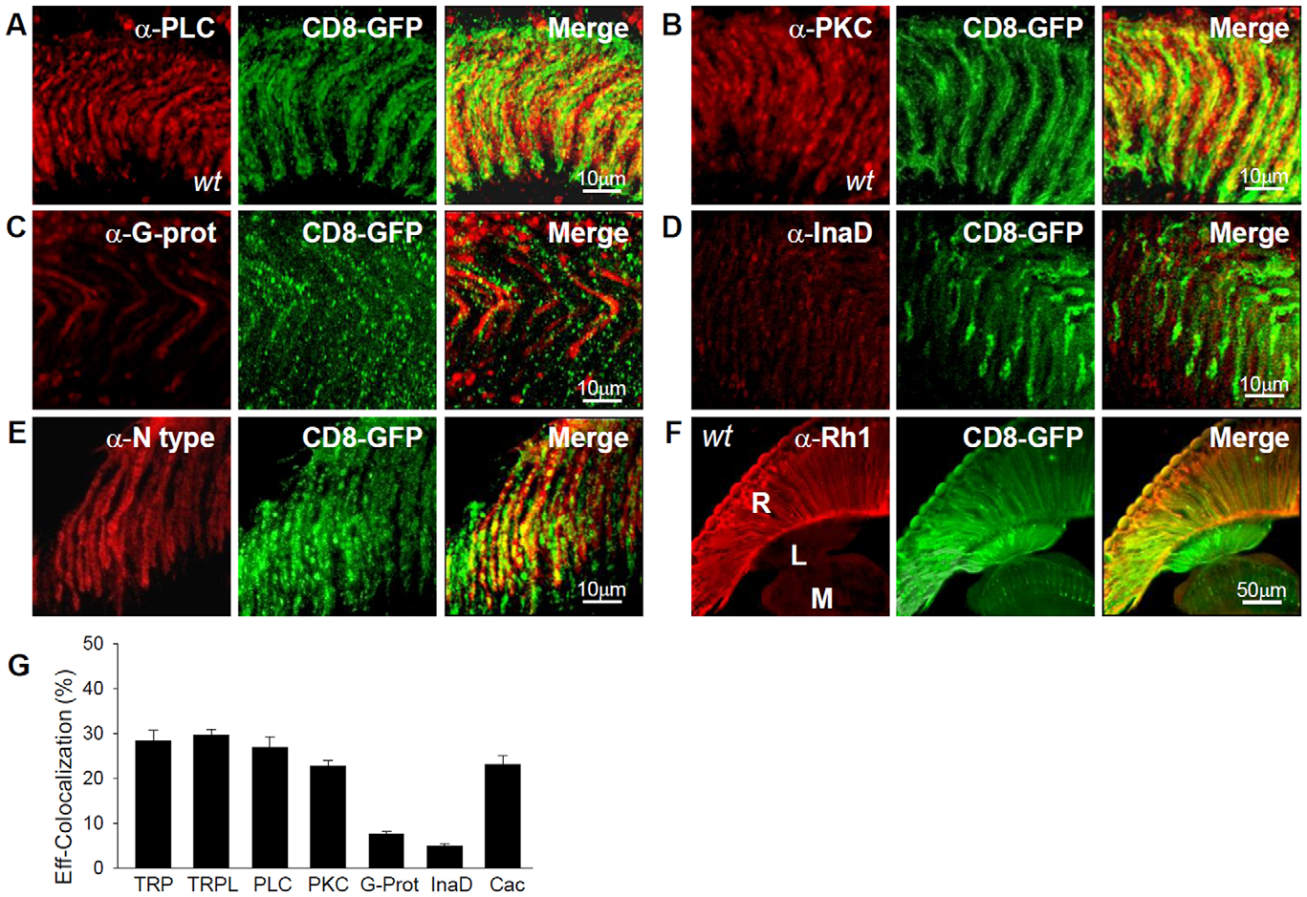
Expression of transduction proteins in photoreceptors axons in the lamina. Z-projections of 10 confocal optical sections (Δz = 0.3 mm) of the lamina showing immunoreaction of antibodies against various proteins in *wt* brain slices. (**A**) Phospholipase C (PLC, left); CD8::GFP (center); Merge (right). (**B**) Protein kinase C (PKC, left); CD8::GFP (center); Merge (right). (**C**) G_q_-Protein subunit (right); CD8::GFP (center); Merge (right). (**D**) InaD (left); CD8::GFP (center); Merge (right). (**E**) Cacophony (left); CD8::GFP (center); Merge (right). *(F)* Rhodopsin (Rh1; left); CD8::GFP (center); Merge (right). (**G**) Effective colocalization of TRP, TRPL, other transduction proteins and cacophony with photoreceptor axons (CD8::GFP) in the lamina. The segmented signals for the different proteins were randomized within the confined regions of photoreceptor axons as described in Methods and illustrated in Supporting Figure S2B. A maximum displacement radius of 20 pixels was considered for randomization. n>20 images for all the immunostainings studied.

To carry out our study, we developed a novel preparation of *Drosophila* brain slices suitable for functional and immunohistochemical studies in the visual system (Fig. 1A,B). Remarkably, this preparation retained the ability to respond to light, manifested as vesicle exocytosis in the lamina. We observed a light-induced decay in FM4-64 fluorescence in preloaded axonal varicosities (Fig. 1C–E; see below and SI for details on bouton quantification). Consistent with a previous report [17], we detected TRP immunoreactivity in the retina and lamina of *wt* flies (Figs. 2A,C, left; n = 12). This was observed by two different monoclonal antibodies directed against the C-terminal region of this channel, with indistinguishable results (Fig. 2E). We also found high immunolabeling for TRPL channels (monoclonal antibody directed against the C-terminal). Thus, both light-dependent channels are present in the same regions of the visual system (Fig. 2B,D, left; n = 10). The specificity of α-TRP and -TRPL antibodies was verified in *trpl^302^*; *trp^343^* null double mutants, where no significant staining was detected (Fig. 2F; n = 6). We examined whether TRP and TRPL immunoreactivity in the lamina corresponded to photoreceptor axons projecting to this neuropile. These axons were identified by ectopic membrane tagged GFP expression (UAS-CD8::GFP) under the GMR-Gal4 driver. In the lamina of adult flies, GMR drives the expression of reporter proteins in the axons of all photoreceptors (R1–R8) [21]. We evaluated whether this marker displayed the same distribution as TRP and TRPL (Fig. 2A–D, center). For dense fluorescence patterns, the degree of random overlap has to be considered for each single colocalization experiment. We applied the confined displacement algorithm (CDA) to assess the colocalization of proteins in small structures, [22], [23]. The CDA allows the evaluation of random colocalization which is subtracted from the Manders colocalization coefficient. This “effective colocalization” therefore corrects for random colocalization and provides an estimate of protein colocalization beyond the random level (see Methods and Text S1). Figure S1 shows a representative example for colocalization analysis of α-TRP and GFP signals, including the ROIs for CDA determined after image deconvolution and segmentation (see Methods). Our results indicate that TRP and TRPL colocalize significantly with GFP (∼30% above the random colocalization; Fig. 3G), indicating that these channels are preferentially distributed within the photoreceptors axons, and perhaps in other cell types as well.

We explored the presence of other key phototransduction proteins in this neuropile, in order to examine a possible functional role in TRP/TRPL activation in the photoreceptor axons. We found a high level of PLC and PKC immunoreactivity (Figs. 3A,B), which colocalized significantly with CD8::GFP (∼25–30% each, above the random values; Fig. 3G). These results show that PLC, PKC, TRP and TRPL are present in photoreceptor axons. In addition, positive immunostaining was found in other axons that we did not further identify. In contrast, we observed a weak immunostaining for G_q_-protein and InaD in photoreceptor axons (∼4–7% above the random values, respectively; n = 6; Figs. 3C,D,G). Rhodopsin (Rh1) immunostaining was confined to the retina, with no detectable presence in the lamina (Figs. 3F; n = 3). Altogether, these results raise the possibility that TRP and TRPL activation in the lamina is mediated by a mechanism involving PLC and PKC.

If present in the synaptic terminals, TRP and TRPL may participate in exocytosis because they permeate Ca^2+^. It is reasonable to expect that they may coexist with a voltage-dependent Ca^2+^ channel. Cacophony is the only fly homologue of vertebrate N-, P/Q- and R-type voltage-gated Ca^2+^ channels and shares ∼68% of its amino acidic sequence with the N-type Ca^2+^ [17], the primary subtype involved in neurotransmission. We explored if cacophony is present in the lamina by testing antibodies against T-, L-, R-, P/Q- and N- subtypes (no specific antibody for cacophony is available). We found positive immunoreactivity exclusively for the α-N-type antibody in the lamina (Fig. 3E, n = 4), where it displayed a significant colocalization of ∼30% above the random value with GFP (Fig. 3G). These results suggest that cacophony is present in photoreceptors axons at the lamina. This possibility is supported by the functional assays described below.

### Cacophony-dependent Ca^2+^ Signaling in Photoreceptor Axons

**Figure 4:**
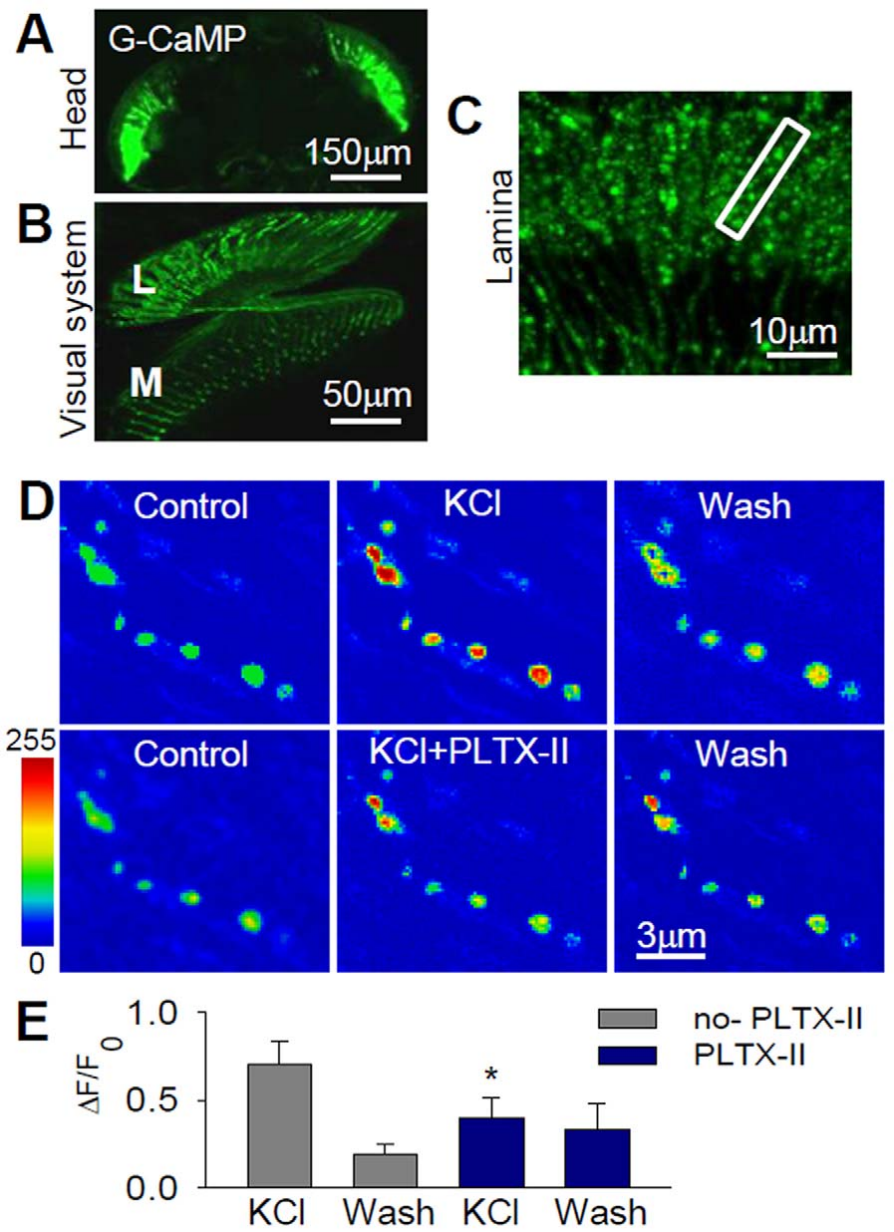
GCaMP-Ca^2+^ fluorescence from photoreceptor axons in the lamina. (**A–C**) Confocal images of the visual system from GMR-Gal4/UAS-G-CaMP transgenic flies showing the expression pattern of the Ca^2+^ indicator protein, G-CaMP, in *Drosophila* photoreceptors. (**D**) Pseudocolor fluorescence images illustrating Ca^2+^ increments upon depolarization induced by high-K^+^ (90 mM); effect of the cacophony blocker PLTX-II on G-CaMP/Ca^2+^ fluorescence changes evoked by high-K^+^. (**E**) Quantification of the G-CaMP/Ca^2+^ fluorescence changes illustrated in (D).

The slices were also amenable for physiological experiments. We first examined whether cacophony channels are implicated in depolarization-induced Ca^2+^ entry in the photoreceptor axons in the lamina. To measure Ca^2+^ signals, we ectopically expressed the fluorescent Ca^2+^-indicator protein G-CaMP [24] under the GMR driver. GMR-Gal4/UAS-G-CaMP flies expressed G-CaMP in photoreceptor somata and axons (Fig. 4A,B); single varicosities were resolved in detail at high magnification in the lamina (Fig. 4C).

In slices from GMR-Gal4/UAS-G-CaMP flies, depolarization by high-K^+^ induced a mean absolute increase of 0.68±0.11 in G-CaMP fluorescence (Fig. 4D,E; n = 4). These responses were significantly reduced by 100 nM of the spider toxin PLTX-II (0.38±0.90, n = 5; Fig. 4D,E), a cacophony channel blocker [25]. These results suggest that cacophony is involved in Ca^2+^ influx in photoreceptor axons.

### Synaptic Vesicle Exocytosis in the Lamina

The immunohistochemical and Ca^2+^ imaging studies described above suggest that cacophony is involved in exocytosis. To examine this possibility, we monitored FM4-64 loading into axonal varicosities as an indicator of previous vesicle release. Bouton quantification (see Methods) was done in an x,y,z volume of 36×36×3 mm^3^. Gradient and size filters were applied to z-projections of 10 images (Δz = 0.3 mm) to create binary images (Figure S2A,B), where the ROIs were automatically quantified (see Methods). The size filter for bouton quantification was set to consider the most representative bouton population (in terms of area) in the lamina, as shown by the distribution of the FM4-64 loaded-boutons in slices from *wt* flies (0.5 to 1.1 µm^2^; Fig. S2C). This procedure was systematically used to quantify boutons in an unbiased manner.

**Figure 5:**
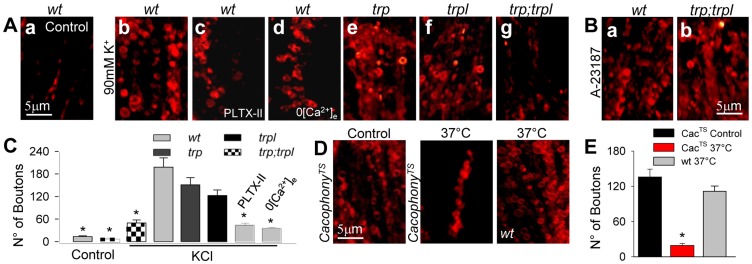
Exocytosis in the lamina depends on TRP, TRPL and cacophony. (**A**) Z-projections of 10 confocal optical sections (Δz = 0.3 mm) showing the fluorescence of FM4-64-loaded boutons in the lamina from: (**a**) *wt* not exposed to high-K^+^ (control); (**b**) *wt* after depolarization by high-K^+^; (**c**) *wt* depolarized by high-K^+^ in the presence of PLTX-II (100 nM); (**d**) *wt* depolarized by high-K^+^ in 0-Ca^2+^ external solution; (**e**) *trp* depolarized by high-K^+^; (**f**) *trpl* depolarized by high-K^+^; (**g**) *trpl;trp* depolarized by high-K^+^. (**B**) Bouton labeling induced by the Ca^2+^ ionophore, A-23187 (250 nM) in *wt* (left) and *trpl;trp* (right) in regular Ringer (5 mM K^+^). (**C**) Quantification of the number of labeled synaptic boutons for the different conditions shown in A. (**D**) Bouton labeling induced by high-K^+^ in slices from *cac^TS^* mutants pre-incubated in chilled Ringer (left, Control) or at 37°C (center) for 10 minutes. Right: bouton labeling at 37°C in slices from wt flies. **(E)** Quantification of the number of synaptic boutons for the conditions shown in D. Bars: mean ± SEM, calculated from z-projections of 10 images. Size: x/y/z = 36/36/0.3 µm^3^. * p<0.05: respect to *wt* high-K^+^ labeling.

In *wt* slices, high-K^+^ pre-treatment in the presence of FM4-64 in chilled Ringer induced massive exocytosis, as revealed by the large number of fluorescently labeled boutons observed in the lamina (199±22; n = 17, Fig. 5Ab,C). Spontaneous vesicle release was estimated in unstimulated slices exposed to FM4-64 in normal extracellular solution in the dark for 10 minutes (control). Compared to high-K^+^ exposure, the number of fluorescently labeled boutons observed in these conditions (20±3, n = 5; Fig. 5Aa,C) was small, confirming that indicator uptake by the boutons was mainly activity-dependent. Once loaded with FM4-64 (Fig. S3Aa), re-exposure to high K^+^ for 5 minutes induced a dramatic decrease in fluorescence, an indication of a massive unloading of boutons preloaded with FM4-64 (Fig. S3), directly demonstrating a strong exocytosis in response to depolarization. Altogether, these results show that the preparation is suitable for recording activity-dependent vesicle exocytosis in the lamina. To evaluate a role of cacophony in exocytosis, we induced depolarization with high-K^+^ in a solution containing PLTX-II and FM4-64. Under these conditions, the number of labeled boutons in *wt* slices was significantly reduced (47±8; n = 7; p<0.01; Fig. 5Ac,C), supporting the idea that this channel is implicated in exocytosis. To further investigate this possibility and considering that PLTX-II could also affect other Ca^2+^channels [26], we took advantage of the cacophony thermosensitive mutant *cac^TS^* [27]. At permissive temperature (chilled Ringer) the number of labeled boutons observed after depolarization by high-K^+^ was 136±11 (n = 6; Fig. 5Da,E). In contrast, in slices incubated at the non-permissive temperature (37°C) for 10 minutes, exocytosis was dramatically reduced (21±9, n = 8; p<0.01; Fig. 5Db and 5E; representing a reduction of 85%). To assess a possible effect of high temperature exposure during exocytosis, *wt* flies were loaded with FM4-64 by high-K+ at 37°C for 10 min. The number of boutons loaded in *wt* in these conditions (112±14, Fig. 5D, right and 5E) was in fact reduced by 45% compared to chilled Ringer (199±22; n = 17, Fig. 5Ab,C), indicating a temperature effect. However, this temperature-dependent reduction cannot account for the 85% decrease observed in *cac^TS^* for the same temperature change, thus confirming that impaired cacophony channel function causes a significant decrease in exocytosis. Consistent with the requirement of Ca^2+^ influx from the extracellular space, experiments conducted in the absence of external Ca^2+^ revealed a dramatic decrease in vesicle loading (36±5 labeled boutons, n = 11; Fig. 5Ad,C). Taken together, these results show that cacophony is necessary for vesicle exocytosis in the lamina.

Our observations that TRP and TRPL channels are also present in the photoreceptor synaptic terminals raised the question of whether these Ca^2+^-permeable channels may also contribute to vesicle release. To address this issue, we evaluated FM4-64 loading after high-K^+^-induced depolarization in synaptic terminals of the lamina in TRP/TRPL mutants. In TRP (*trp*) and TRPL (*trpl*) single mutants the level of exocytosis induced by depolarization was comparable to *wt* flies (150±19 labeled boutons, n = 7, Fig. 5Ae,C and 122±16, n = 9, Fig. 5Af,C, respectively; p>0.05). In contrast, a severe impairment in vesicle release was observed in *trpl*; *trp* double mutants (49±9 labeled boutons, n = 22, p<0.01; Fig. 5Ag,C). The basal bouton loading observed in non-depolarized double mutant slices was significantly lower than under depolarization (17±5, n = 5; p<0.01; Fig. 5C). These results are consistent with the presence of a voltage-dependent Ca^2+^ influx in the terminals, independent of TRP and TRPL.

It was conceivable that the inability of *trpl;trp* synaptic terminals to undergo exocytosis could be caused by degeneration of the vesicle release mechanism. To test for this possibility, the Ca^2+^ ionophore A-23187 (250 nM) was added to the external solution in the presence of FM4-64. This treatment induced vigorous vesicle release, being the number of labeled boutons in *trpl;trp* flies similar to *wt* in the same conditions (90±18; n = 5 and 130±15; n = 5; Figs. 5Bb and a, respectively). This result indicates that the failure of the double mutant to undergo exocytosis relied on the absence of the Ca^2+^-permeable channels, TRP and TRPL, rather than on a morphological defect of the terminals. Interestingly, the presence of either TRP or TRPL is sufficient to sustain synaptic exocytosis.

Altogether, these results are consistent with the hypothesis that TRP, TRPL and cacophony are involved in vesicle release in the lamina. However, the fact that exocytosis is almost completely abolished by removal of either cacophony or of both, TRP and TRPL is intriguing. In the experiments described in the following sections, we aimed to unravel the mechanism of neurotransmitter release in photoreceptor terminals and to clarify the relative contribution of these different types of Ca^2+^-permeable channels to this process.

### Contribution of Intracellularly Liberated Ca^2+^ to Exocytosis

**Figure 6:**
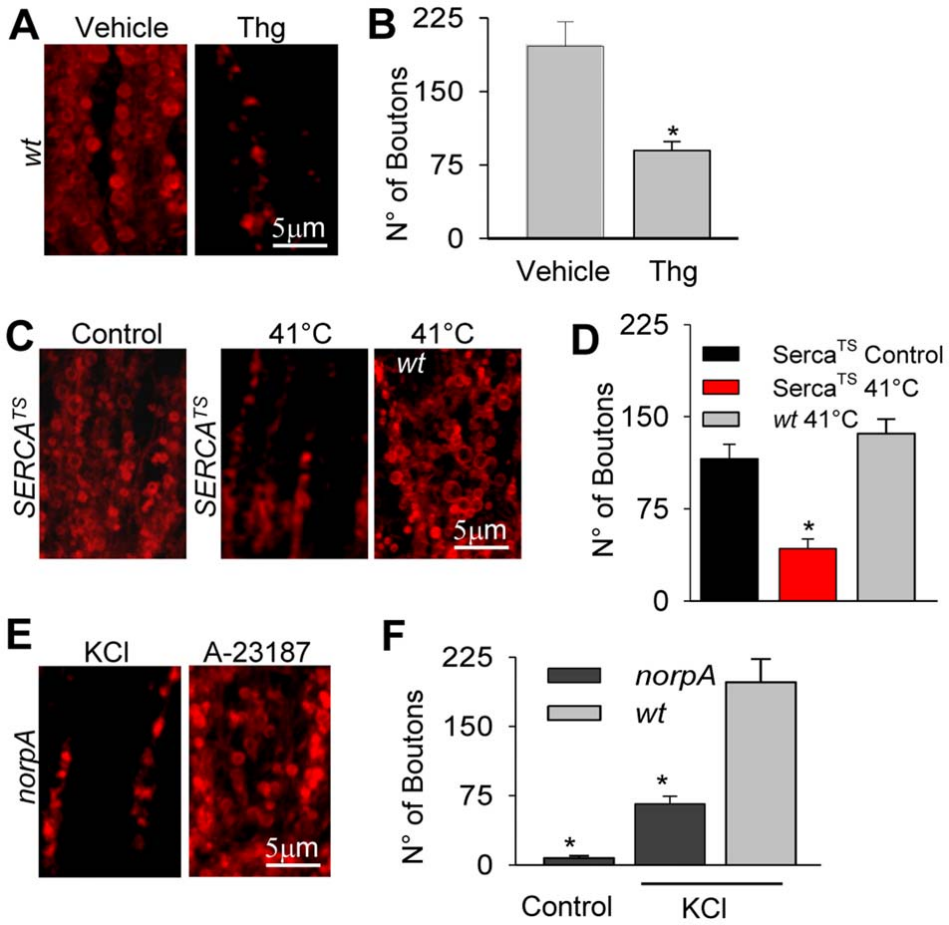
Intracellular Ca^2+^ stores and PLC contribute to exocytosis in the lamina. Z-projections of 10 confocal optical sections (Δz = 0.3 mm) showing representative confocal images from slices loaded with FM464. (**A**) *wt* slices pre-treated with Thg (10 µM; right) or its vehicle (DMSO; left), and depolarized with high-K^+^. (**B**) Quantification of the number of synaptic boutons observed for the conditions in A. (**C**) Bouton labeling induced by depolarization in slices from *dserca^TS^* mutants pre-incubated in chilled Ringer (Control; left) or at 41°C (center) for 2 minutes during depolarization; an example of labeling at 41°C in slices from wt flies is also shown (right). (**D**) Quantification of the number of synaptic boutons for the conditions in C. (**E**) Slices from the PLC mutant *norpA* were depolarized with high-K^+^ (left) and treated with A-23187 in normal Ringer (5 mM K^+^; right). (**F**) Quantification of the number of synaptic boutons loaded by depolarization in *norpA* and *wt* slices. Control: basal loading in *norpA* in the absence of depolarization. Bars: mean ± SEM, calculated in z-projections of 10 images. Size: x/y/z = 36/36/0.3 µm^3^. * p<0.05: with respect to *wt* treated with high-K^+^.

We investigated whether Ca^2+^ provided by the ER was relevant for vesicle release by depleting this organelle using pharmacological and genetic disruption of Ca^2+^ uptake. This process relies on the ER Ca^2+^-ATPase (SERCA), which is strongly expressed in the lamina (26). The *Drosophila serca* gen has about 70% identity with the mammalian *serca1*, with which it shares identical binding sites for its inhibitor thapsigargin (Thg) [28]. Wild type slices pretreated with Thg (10 µM) in Ringer exhibited a 52% reduction in bouton labeling with depolarization compared to pre-treatment with the vehicle (DMSO; 87±9, n = 7, vs. 182±14 boutons; n = 5; p<0.01; Fig. 6A,B). These results support the notion that Ca^2+^ released from the ER contributes to synaptic activity in the lamina. As an independent test for the involvement of ER Ca^2+^ in exocytosis, we used the thermosensitive mutant (*serca^TS^*) [28]. In slices incubated at non-permissive temperature (41°C) for 2 minutes, depolarization-induced exocytosis was reduced 64% as compared to permissive temperature (chilled Ringer, 42±7, n = 7, vs. 41°C, 117±9, n = 5; p<0.01; Fig. 6C,D). When slices from *wt* flies were loaded with FM4-64 at 41°C for 2 minutes, the number of fluorescently labeled boutons (136±12, n = 6; Fig. 6C,D) was reduced by only 32% compared to chilled Ringer, a temperature effect that does not explain the 64% reduction in mutants at non-permissive compared to permissive temperature. These results indicate that intracellularly released Ca^2+^ is necessary for normal vesicle exocytosis at the lamina.

### Regulation of Exocytosis by PLC

The immunohistochemical stainings presented above suggested that PLC is present in the photoreceptors axons, where it may participate in exocytosis by activating TRP and TRPL. We investigated this by examining synaptic bouton labeling in the hypomorphic PLC mutant, *norpA^P24^*. There was depolarization-induced FM4-64 loading in this mutant (66±9, n = 8), as compared to *norpA* not exposed to high-K^+^ (control; 8±3, n = 5; p<0.05; Fig. 6F), although the number of fluorescently labeled boutons by depolarization was significantly lower in the mutant than in *wt* (66±9, n = 8, vs. 199±22, n = 17; p<0.05; Fig. 6E,F), implicating PLC. We confirmed the integrity of the synapse in *norpA* by adding A-23187, which induced massive exocytosis (168±23 labeled boutons, n = 4; Fig. 6E, right). This result suggests that PLC is involved in depolarization-induced synaptic activity in the lamina.

### Ca^2+^ Influx Mediated by TRP and TRPL as Store-operated Channels

**Figure 7:**
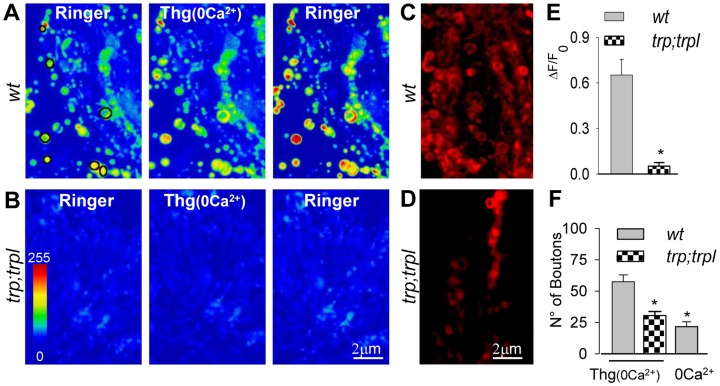
Ca^2+^ signals induced in the lamina by the ‘Ca^2+^ depletion protocol’ are abolished in the *trpl;trp* mutant. Slices were bathed with 0-Ca^2+^ solution supplemented with 10 µM Thg (Thg(0Ca^2+^)) during 8 min to produce internal Ca^2+^ stores (ER) depletion. Afterwards the slices were returned to regular Ringer. (**A, B**) Z-projections of 4 confocal images were obtained from the lamina in slices preloaded with Rhod-2 and treated with the depletion protocol. Rhod-2 fluorescence in *wt* (A) or *trpl;trp* (B) slices in regular Ringer (left), during application of Thg(0Ca^2+^) solution (center) and after regular Ringer was restored (right). (**C–D**) Z-projections of 10 confocal optical sections (Δz = 0.3 mm) showing representative bouton loading of FM4-64 in *wt* (C) and *trpl;trp* (D) slices, upon returning to regular Ringer after ER depletion. (**E**) Quantification of Rhod-2 fluorescence change after restoring Ringer in *wt* (A) and *trpl;trp* (B). (**F**) Quantification of FM4-64 bouton labeling induced by the depletion protocol (C and D, denoted by “0Ca^2+^+Thg”) or upon returning to Ringer after treatment with 0-Ca^2+^ solution without Thg (“0Ca^2+^”). Bars: mean ± SEM calculated in z-projections of 10 images. Size: x/y/z = 36/36/0.3 µm^3^. * p<0.05 with respect to *wt.* Pseudocolor scale in arbitrary units.

Considering that intracellular Ca^2+^ release and that TRP/TRPL contribute to vesicle release in the lamina, we examined the possibility that these channels function as store-operated channels (SOCs) in the photoreceptors axons. We utilized a well-established experimental protocol to test the participation of SOCs, known as the “Ca^2+^-depletion protocol” [29]. In this protocol, the Ringer solution is exchanged by a Ca^2+^-free (0-Ca^2+^) Ringer supplemented with Thg (10 µM) to deplete the ER of Ca^2+^, a condition that should open SOCs, if present. If a subsequent restitution of regular Ringer induces a transient Ca^2+^ influx, it is taken as an indication of SOC activity. In GMR-Gal4/UAS-G-CaMP flies, this protocol induced a significant increase in Ca^2+^-dependent GCaMP fluorescence in the lamina (mean absolute change  = 0.38±0.06, n = 5; not shown). This result is consistent with the existence of a SOC mechanism in photoreceptors terminals in the lamina. Considering the difficulty to express G-CaMP in *trpl;trp* flies due to genetic constraints (see Methods), we carried out the same experiment in the *wt* and in the double mutant using the Ca^2+^ indicator Rhod-2 AM. In *wt* slices loaded with this fluorophore, the Ca^2+^-depletion protocol induced a mean absolute increase in fluorescence of 0.67±0.09, (n = 4; Fig. 7A,E). In contrast, no change in Rhod-2 fluorescence was observed in *trpl;trp* slices (0.06±0.03, n = 4; Fig. 7B,E).

It might be thought that Ca^2+^ release caused by SERCA inhibition activates PLC [28], thereby leading to the opening of TRP/TRPL, without involving a SOC mechanism. Thg (0-Ca^2+^) exposure should actually increase cytosolic Ca^2+^, however after 10 min in this solution, internal Ca^2+^ should be back to original level [29]. Accordingly, there was no significant difference in fluorescence between this condition (Fig. 7A, center) and the initial condition (Fig. 7A, left; mean absolute change = −0.04±0.03; n = 5).

Altogether, these results suggest that TRP and TRPL activation in the photoreceptor axons in the lamina is mediated by a store-operated mechanism.

We next investigated if the Ca^2+^ increase associated with TRP/TRPL SOC activity was able to evoke exocytosis in the varicosities. Slices from *wt* flies were exposed to Thg in 0-Ca^2+^ external solution for 10 min and then returned to normal Ringer supplemented with FM4-64. If the depletion protocol triggers exocytosis, the dye will be subsequently incorporated into axonal varicosities. As shown in Fig. 7C,F a significant number of boutons was labeled by this procedure (58±7, n = 7). To check if exposure to 0-Ca^2+^ Ringer could be by itself responsible for the exocytosis observed upon return to regular external solution, we repeated the experiment in the absence of Thg. In this case, the number of loaded boutons (24±7, n = 5, p<0.05; Fig. 7F) was significantly smaller than when loaded by the Ca^2+^-depletion protocol and comparable to control loading in Ringer, without depolarization (20±3, n = 5; see Fig. 5C). This result supports the presence of Ca^2+^-permeable SOC channels and that they represent an additional source of Ca^2+^ for the synaptic events occurring in the varicosities.

Importantly, in the *trpl;trp* mutant the Ca^2+^-depletion protocol failed to induce vesicle exocytosis, as revealed by the low number of labeled boutons observed (29±4, n = 6; Fig. 7D,F), that was comparable to basal bouton loading in these mutants (17±5, n = 5; Fig. 5C). As previous experiments with the Ca^2+^ ionophore (A-23187) demonstrated that the double mutant has the potential for vesicle release (Fig. 5B), the impaired exocytosis induced by the depletion protocol in these slices points to a SOC function of TRP and TRPL.

Altogether, these results indicate that there is a Ca^2+^ influx through TRP and TRPL channels localized to the axons. These channels appear to be activated by a store-depletion mechanism and may contribute to Ca^2+^-dependent vesicle exocytosis.

It is remarkable that TRP and TRPL function differently in the rhabdomere and in the axonal terminals of the photoreceptors, where they appear to be opened by different mechanisms.

## Discussion

TRP and TRPL are the targets of *Drosophila* phototransduction in the rhabdomere, gated by an as yet undetermined PLC-dependent mechanism independent of internal membrane systems, which are absent in the microvilli. Here we provide the first evidence that both channels additionally participate in exocytosis in photoreceptor synaptic terminals, where they can be activated by depletion of Ca^2+^ stores. We also demonstrate that the voltage-dependent Ca^2+^ channel, cacophony, plays a critical role in exocytosis.

### Presence of TRP, TRPL, Other Phototransduction Proteins and Cacophony in the Lamina and Medulla

We confirmed that, in addition to the rhabdomere, TRP localizes to the lamina and the medulla [17]. Additionally, we found TRPL in these two neuropiles, where photoreceptors synapse with secondary neurons. We studied the lamina, where most photoreceptors make synaptic connections into well-defined structures [3].

We developed a *Drosophila* slice preparation suitable for immunohistochemistry and functional experiments in the lamina. In addition to TRP and TRPL, PLC and PKC exhibited high expression levels, while G_q_ and INAD were scarce and rhodopsin was absent. The four former proteins colocalized with ectopically expressed GFP, used as photoreceptor marker, whereas G_q_ and INAD colocalization with GFP was low. While TRP, TRPL and PLC were not restricted to photoreceptors, the relevant conclusion is that their presence in photoreceptors axons in the lamina suggests a participation in presynaptic events.

The prominent cacophony immunostaining in the lamina is relevant. This Ca^2+^ channel is involved in synaptic transmission in *Drosophila* neuromuscular junction, brain and retina [19], [30]. Cacophony mutants ERGs show abnormal ‘on-off’ transients [31], [32], suggesting a role in synaptic transmission in the lamina.

### Evidence for a Synaptic Role of Cacophony, TRP and TRPL

A role of cacophony in photoreceptor synaptic transmission is supported by our observation that inhibition of this channel by PLTX-II affected bouton labeling. Although the possibility that PLTX-II could also affect other Ca^2+^ channels cannot be ruled out [26], the role of cacophony in vesicle release was further strengthened by the substantial reduction in FM4-64 fluorescence in the thermosensitive cacophony mutant *cac^TS^* at non-permissive temperature. In agreement with this, a mutation in the *dα_2_δ-3* gene encoding a cacophony subunit abolishes the ERG ‘on` transient [31]. On the other hand, depolarization–induced G-CaMP Ca^2+^ fluorescence changes in the photoreceptors were significantly decremented by PLTX-II, providing additional evidence involving cacophony in the synaptic events.

The observations that TRP and TRPL are also in the photoreceptors axons and are considerably Ca^2+^-permeable (P_Ca_:P_Na_ ∼100∶1 and ∼4∶1, respectively) [8] suggested a synaptic role. Accordingly, vesicle release was drastically impaired in the double mutant. Opening a Ca^2+^ pathway with the ionophore induced exocytosis in this mutant, an observation that opposes to a generalized degeneration of synaptic machinery. This evidence shows that TRP and TRPL are involved in exocytosis. Only one of these channels was sufficient for sustaining exocytosis.

FM4-64 is presumably incorporated by all lamina neurons and therefore not only photoreceptor boutons should be labeled. However, we expect that the dramatic changes in release observed include photoreceptor terminals, which represent the most numerous synaptic contacts in the lamina [33].

Altogether, our results support the participation of TRP, TRPL and cacophony in synaptic transmission in photoreceptor terminals.

### Role of Phospholipase C and Internal Ca^2+^ Reservoirs in Synaptic Transmission

What is PLC doing in photoreceptors synaptic terminals? Depolarization-induced exocytosis was markedly reduced in *norpA* mutant, suggesting a role of PLC in neurotransmitter release. An obvious possibility is that it mediates TRP/TRPL activation. In principle, PLC may act by either DAG or IP_3_. PUFAs can activate the light-dependent channels when added to intact ommatidia [9], as well as to excised rhabdomeric membrane patches, in which DAG can do the same [10]. Thus, it is conceivable that these lipids may also activate TRP/TRPL channels in the lamina. Nevertheless, there is no evidence that PUFAs are generated in these photoreceptors.

How is PLC activated? In *Drosophila* photoreceptors, a level of PLC activity has been observed both *in vitro* and in *vivo* [28], [34]. This basal activity is probably a property of the PLC molecule itself, as it is not affected by mutation of G_q_-protein [34]. In addition, a positive modulation of PLC activity by micromolar Ca^2+^ has been reported in *Drosophila* head membranes [28]. Therefore, basal PLC activity could be boosted by Ca^2+^ influx through cacophony (and additional Ca^2+^ pathways described here) during depolarization-induced vesicle exocytosis, representing a feed-forward mechanism in this graded synapse. Alternatively, PLC activation may be a consequence of a direct activation of G_q_ by depolarization, as reported in other insects [35]. On the other hand, the substantial PKC expression in the terminals suggests that it may down-regulate PLC, as in the rhabdomere [36].

Calcium reservoirs appear to be involved in exocytosis, since inhibition of SERCA with Thg deeply affected vesicle release. Moreover, exposure of *serca^TS^* to the non-permissive temperature considerably decreased bouton labeling compared to permissive temperature, and we showed that this decrease cannot be explained exclusively by a temperature effect. These results strongly implicate ER Ca^2+^ release in photoreceptors exocytosis.

### TRP and TRPL as SOCs in Photoreceptor Synaptic Terminals

The robust Ca^2+^ signals in the lamina after Ca^2+^ depletion implicated TRP/TRPL, as it was absent in *trpl;trp* animals. This supports the function of TRP/TRPL as SOCs in the synaptic terminals, allowing Ca^2+^ influx. This mechanism drives exocytosis, as indicated by the Ca^2+^-depletion protocol, where bouton labeling was significant. Interestingly, TRP and TRPL function as SOCs in heterologous expression systems [13], but not in the rhabdomere [14], [15].

Mammalian homologues of *Drosophila* TRP, TRPC1, 2, 4 and 6, are proposed to function as SOCs in different cell types [37]. Moreover, TRPC1 operating as SOC regulates Ca^2+^ influx related to neurotransmission in rods and cones [38]. The *Drosophila* genome has one gene encoding STIMh [39], an ER Ca^2+^ sensor protein that forms functional SOCs in association with TRPC1 [40]. It remains to be determined whether TRP/TRPL could form equivalent presynaptic macromolecular complexes in photoreceptors.

We showed that it is improbable that in the Ca^2+^-depletion experiments TRPL/TRP opening could be induced by a PLC-dependent mechanism mediated by phospholipase activation by a cytoplasmic Ca^2+^ increase due to altered reticular release/uptake balance during Thg treatment. In these experiments PLC contribution to exocytosis was possibly by-passed. In normal conditions, this enzyme may elicit Ca^2+^ elevation in the synaptic terminals by DAG-mediated activation of TRP/TRPL and/or by inducing Ca^2+^ release.

### Diversity of Ca^2+^ Sources in Photoreceptor Synaptic Terminals: Functional Implications

Photoreceptors synaptic transmission must accurately follow the fast photoresponses generated in the rhabdomere. As graded synapses support rapid changes in neurotransmitter release, they should undergo fast variations in internal free Ca^2+^ [41]. Small and fast Ca^2+^ increments induce correspondent changes in release, something that would be implausible if a threshold were involved, as in non-graded synapses.

Besides cacophony contribution to exocytosis, the presence of the ryanodine receptor (RyR) in the lamina [42] suggests the participation of Ca^2+^-induced Ca^2+^ release (CICR), but we lack direct evidence for this. CICR regulates exocytosis in rods allowing high rates of neurotransmitter release [43]. A reasonable expectation is that *Drosophila* photoreceptors use all available Ca^2+^ pathways (cacophony; TRP/TRPL; the IP_3_ receptor, IP_3_R and RyR) to satisfy the synaptic demands required by their extremely fast photoresponses [6]. Weckström et al [44] speculated that the IP_3_R might reinforce transmitter release, but showed no direct evidence for it. This possibility is supported by our results implicating PLC. Moreover, our observation that Ca^2+^ from the ER contributes to depolarization-induced exocytosis strengthens the possibility of internal release via IP_3_R and/or RyR.

Bouton labeling experiments were conducted under prolonged depolarization, implying that vesicle exocytosis was at steady-state. Thg experiments under such conditions show that released Ca^2+^ plays an essential role in neurotransmission. In tonic synapses, this mechanism may be crucial to sustain synaptic transmission for extensive periods of time.

**Figure 8:**
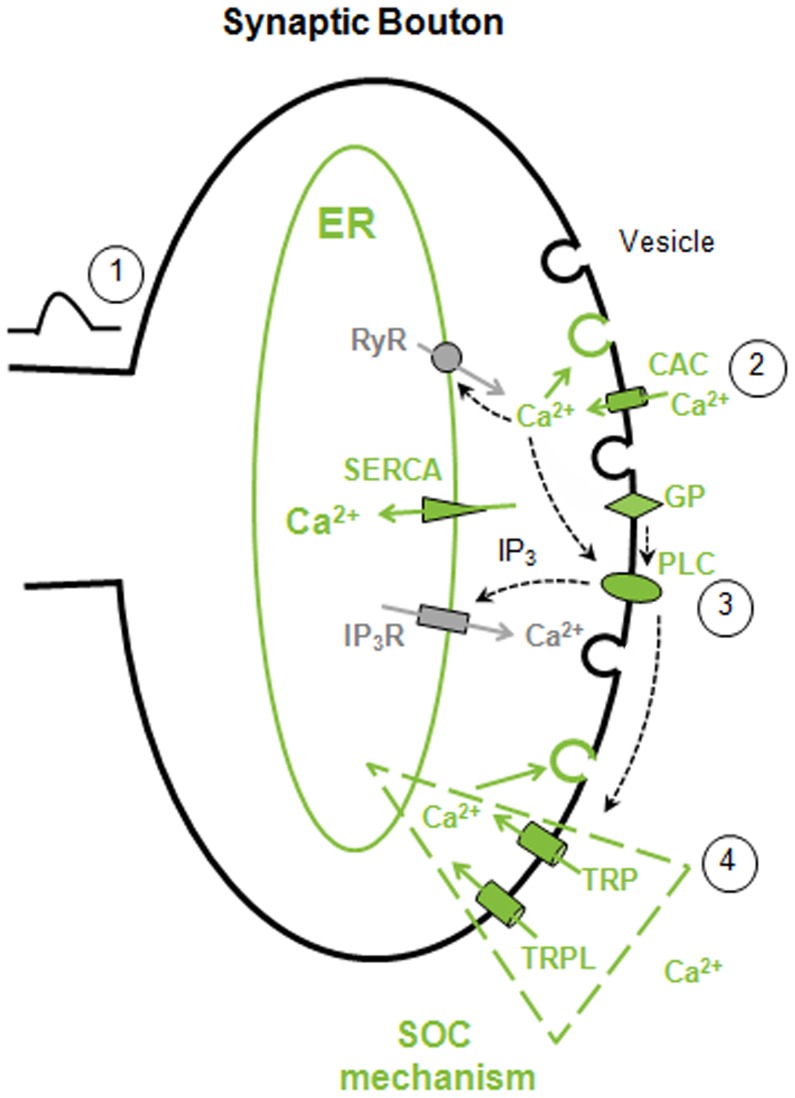
Model for photoreceptor synaptic events in the lamina. A graded depolarization from the soma reaches the axon terminals (1) opening cacophony, which allows the Ca^2+^ influx (2) that triggers exocytosis. Additional mechanisms complement or amplify the Ca^2+^ signal: Ca^2+^ release from the endoplasmic reticulum (ER) (3), PLC activation (4) and Ca^2+^ influx through TRP/TRPL channels (5). Internal Ca^2+^ release could be due to ryanodine receptor (RyR) activation, a mechanism termed Ca^2+^-induced Ca^2+^ release, or mediated by IP_3_ receptor (IP_3_R) opening as a result of PLC activity. TRP/TRPL working as store-operated channels (SOCs) contribute Ca^2+^ to exocytosis and could also be modulated by PLC-dependent lipid changes. The massive raise of Ca^2+^ from these multiple pathways allows extremely fast exocytosis at the synaptic terminal. In green are displayed the components and pathways shown by us to be involved in vesicle exocytosis. The broken lines denote hypothetical pathways.

We propose the following model for the synaptic events at the axon terminals (Fig. 8): the receptor potential activates cacophony in the axon, allowing its propagation towards the axonal terminal, where Ca^2+^ enters through cacophony inducing vesicle release, perhaps enhanced by CICR. Additionally, PLC activated by an unknown mechanism which may be Ca^2+^ itself or depolarization, generates IP_3_, triggering Ca^2+^ release through IP_3_Rs. ER Ca^2+^ depletion in turn opens TRP/TRPL by a SOC mechanism, incrementing the Ca^2+^ supply. These channels may also be opened by lipid and pH changes resulting of PLC activity [13]. This multi-source transient Ca^2+^ increment guarantees efficient, rapid and sustained neurotransmitter release. After depolarization, resting Ca^2+^ levels would be restored by extrusion by the Na^+^/Ca^2+^ exchanger [45] and uptake by the ER.

We think that it is important to integrate the data into a plausible working model that could be helpful for designing further experiments. Although our model accounts for the data, it is by no means the only possible one. Accordingly, some aspects of it may be interpreted differently or given a different weight. For example, the relative contributions of cacophony, CICR, IP_3_-induced Ca^2+^ depletion and TRP/TRPL to presynaptic Ca^2+^ for vesicle release can vary widely. Also, the activation of TRP/TRPL may rely on ER depletion and/or lipids associated to PLC activity. It may be thought that the Ca^2+^ influx through cacophony should be sufficient to account for exocytosis, making Ca^2+^ release redundant and rather unnecessary. However, in this graded synapse the level of cacophony activation will follow the graded depolarization. The amplitude attained by the receptor potential are most likely within a small voltage range above the threshold for cacophony activation (-20 or -40 mV [20], inconsistent with a massive cacophony-dependent Ca^2+^ influx. Therefore, additional Ca^2+^ sources amplifying this initial signal are likely to be required for light-induced synaptic transmission.

We provide novel evidence for TRP/TRPL function in *Drosophila* photoreceptors. For the first time, we show that these channels have dual roles in separate regions of the same cell, namely the rhabdomere and the synapse, apparently involving different mechanisms. More generally, the observations reported herein shed light on the mechanism controlling presynaptic events in graded synapses.

## Materials and Methods

### *Drosophila* Fly Strains

The following *Drosophila melanogaster* strains were used: wild type (*wt*) Oregon Red, *w;trp^343^*, *w;trpl^302^*, *w;cn,trpl^302^;trp^CM^, w;trpl^302^;trp^343^* and *w;norpA*. We confirmed the integrity of the retinae of these flies with the pseudopupil test [46] and electroretinogram. We also used the stocks GMR-Gal4,UAS-mCD8-GFP/Cyo, GMR-Gal4/Cyo, UAS-G-CaMP. The thermosensitive mutants *cac^TS2^* and *dserca^TS^/Cyo* were kindly provided by R.W. Ordway and S. Sanyal, respectively. For Ca^2+^ fluorescence experiments, UAS-G-CaMP flies were crossed to GMR-Gal4/Cyo and the progeny of 2–4 days post-eclosion was utilized. Flies were reared at 18°C under dim light conditions.

### *Drosophila* Brain Slices

Adult male and female flies were anesthetized in CO_2_ and kept in ice for sectioning with a vibratome (Vibratome, 1000 plus) under continuous illumination. For each experiment, around 10 flies were stuck to the stainless steel tray with 1 µL of cyanoacrylate ester glue. Flies were immersed in chilled extracellular solution containing (in mM): 120 NaCl, 5 KCl, 8 MgSO_4_, 1 CaCl_2_, 25 L-proline, 1.25 NaH_2_PO_4_ and 25 NaHCO_3_, 2.5 sucrose, 10 HEPES. This solution was bubbled with a mixture of 95% O_2_-5% CO_2_, which maintained the pH at 7.15. The whole body was sectioned in transversal slices (200 µm thick), yielding one slice per individual. These slices were suitable for both immunofluorescence and functional analysis. In the latter case, slices were kept in iced Ringer in the dark, for at most 2 hours, before imaging. The experiments were carried out at room temperature (approximately 20°C). We considered depolarization-induced FM4-64 or Rhod-2 AM loading into axonal varicosities as a criterion for slice functionality. In mutants where depolarization-induced loading was disrupted, we performed internal positive controls using pharmacological tools to show that the terminals can effectively be loaded. All functional experiments were performed in chilled medium, except for those with the temperature-sensitive mutants at non-permissive temperatures and experiments in wt at 37/41°C to evaluate the effect of temperature.

### Antibodies and Reagents

The α-TRP monoclonal antibody MAb83F6 developed by Seymour Benzer was purchased from the Developmental Studies Hybridoma Bank (DSHB) under the auspices of the NICHD and maintained by The University of Iowa, Department of Biological Sciences, Iowa City. A second α-TRP monoclonal antibody was kindly provided by Craig Montell (Johns Hopkins University); both antibodies were tested for specificity in the double mutant *trpl;trp*. The α-TRPL polyclonal antibody AB5912 was obtained from Chemicon and the pan α-N-type Ca^2+^ α-1B polyclonal antibody (L-17), from Santa Cruz [47]. Polyclonal antibodies directed against PLC, PKC, INAD [48], [7], G_q_-Protein and Rh1 were kindly provided by Charles Zuker (Columbia University). Specificity of the latter four antibodies was previously tested [49]. Alexa-Fluor 546 conjugated goat α-mouse and goat α-rabbit, Texas Red-X phalloidin, Bodipy TR-X thapsigargin, TO-PRO-3 iodide Alexa 633 and the calcium ionophore A-23187 were from Molecular Probes, Invitrogen. The spider toxin Plectreurystoxin II (PLTX-II) was purchased from Alomone Labs.

### Immunohistochemistry

Flies were anesthetized in CO_2_ and fixed for 48 h in 4% formaldehyde in phosphate buffer saline (PBS) at 4°C. 200 µm thick horizontal head sections were cut with a vibratome in chilled PBS under illumination, as described above. The sections were incubated for 2 h at room temperature (20–22°C) in blocking solution containing: 50% goat serum, 10% bovine seroalbumin (BSA) in PBST (PBS +0.1% Triton X-100; Sigma). Primary antibodies were incubated overnight at the appropriate dilution in blocking solution at 4°C (α-TRP 1∶200, α-TRPL 1∶1000, α-G_q_, α-PLC and α-INAD 1∶300). Slices were washed 4×20 min in PBS and incubated with secondary antibody (1∶200) for 1 h at room temperature. Either goat α-rabbit AlexaFluor594 or goat α-mouse AlexaFluor546 conjugated IgG antibodies were used. Sections were then washed (4×20 min) in PBS and stored in glycerol at 4°C. For imaging, slices were enclosed between two coverglasses and immersed in mounted media (Fluoromount, Electron Microscopy Sciences) and 20% glycerol.

### Statistics

For all quantifications we utilized as statistical tests one or two-way ANOVA with Dunnett’s and Bonferroni post-test, p<0.05. Error bars represent ± S.E.M.

### Ca^2+^ Imaging

*Drosophila* slices from GMR-Gal4/UAS-G-CaMP flies were covered with low melting point Agarose (type IX-A, Sigma-Aldrich) to prevent movement. They were constantly perfused with normal extracellular solution (1 mL/min, bubbled with 95% O_2_-5% CO_2_) and a glass micropipette positioned on the lamina was used for local application of high-K^+^ solution (in mM: 35 NaCl, 90 KCl, 8 MgSO_4_, 1 CaCl_2_, 25 L-proline, 1.25 NaH_2_PO_4_ and 25 NaHCO_3_, pH 7.15). Ca^2+^ signals were recorded by the fluorescent protein G-CaMP, ectopically expressed in the photoreceptors of GMR-Gal4/UAS-G-CaMP flies. This approach was not used in *trpl;trp* mutants because in GMR-Gal4 flies the *gal4* construct localizes in the same chromosome as *trpl*. This could be addressed by making GMR-Gal4,*trpl;*trp recombinants and UAS-GCaMP;*trpl;trp* segregant. Since this is a rather demanding and time consuming approach, we circumvented it by measuring Ca^2+^ in *trpl^302^; trp^343^* mutants and *wt* flies with the fluorophore Rhod-2-AM. The slices were incubated for 30 minutes in the dye (10 µM in normal extracellular solution) and then washed for 10 minutes. In the SOC protocol, after Rhod-2-AM loading, Ringer solution was replaced by 0-Ca^2+^ Ringer supplemented with 10 µM Thg for 10 min; after this, normal Ringer was restored. Confocal z-stacks images were acquired before, during and after stimulation. See SI for details on image capture and analysis.

### Vesicle Exocytosis (FM4-64 Imaging)

Vesicle exocytosis in the lamina was evaluated by the membrane fluorophore FM4-64 imaging in brain slices. Synaptic terminals were labeled with FM4-64 (10 mM) in an activity-dependent manner [50]. Discrete boutons were clearly distinguishable in *wt* and mutant flies. Loading procedure was as follows: in darkness, slices were stimulated in ice with high K^+^ extracellular solution (90 mM) in the presence of FM4-64 for 10 minutes and then washed for 5 minutes in normal extracellular solution. In store-depletion experiments, FM4-64 was applied after 8 min of thapsigargin (Thg, 10 µM) pre-treatment and washed out with Ringer. Basal dye labeling was evaluated without stimulation, in the presence of FM4-64 for 10 minutes. Along the paper the notation “control” refers to basal labeling of FM4-64 or other dyes. Only slices with morphological preservation of the lamina were used. See below and SI for details on image acquisition and analysis.

### Image Capture and Analysis

Acquisition of the different types of fluorescence images obtained in this study is described in detail in SI. Raw confocal image stacks were deconvolved by Huygens Scripting software (Scientific Volume Imaging, Hilversum, Netherlands) using an algorithm based on the Classic Maximum Likelihood Estimator. Image-processing routines were developed in SCIAN laboratory (www.scian.cl) based on interactive data language (IDL, ITT, Boulder, Colorado). These procedures were used for ROI segmentation, quantification, and to determine colocalization coefficients (see below).

### Quantification of FM4-64 Labeled Synaptic Boutons

A semi-automated analysis was developed to quantify synaptic boutons in an unbiased manner (see SI for details on gradient and size filters). The selected ROIs were automatically quantified by IDL. Results are expressed as mean number of fluorescently labeled boutons (mean ± standard error, SEM) from several projection images, each from a different fly. One- and two-way ANOVA with Dunnett’s and Bonferroni post-test statistical analysis was performed using GraphPad Prism 4 for Windows (GraphPad Software, San Diego California USA).

### Colocalization Analysis

The confined displacement algorithm (CDA) was performed according to Ramírez et al, [22], segmented cartridges confined the displacement area. Briefly, with correlation techniques it is possible to shift one channel and its corresponding image mask (which confines radial displacement to the defined axonal section) relative to the second channel. Random colocalization of two fluorescent signals in the x/y plane was estimated by calculating its probability of occurrence within the cartridges (M1_ROI_(random), see SI). M1_ROI_(random) was subtracted from the Manders colocalization coefficient calculated without any displacement M1_ROI_(d = 0) and referred to as effective colocalization M1_ROI_(effective). Expressed as a percentage, Eff-Colocalization (%)  =  M1_ROI_(effective) * 100.

## Supporting Information

Figure S1 **Segmentation of ROIs for colocalization analysis by the confined displacement algorithm (CDA).** (**A**) Representative 2-channel confocal image with α-TRP (Ch1, red, left) and mCD8-GFP (Ch 2, green, right). (**B**) Confined region defines the photoreceptors axons (from Ch2) after segmentation (left). Merge of the segmented signals for Ch1 (red) and Ch2 (green) within the confined ROI (grey) (right).
(TIF)

Figure S2 **Bouton detection and quantification in confocal images.** (**A**) Representative image of lamina boutons stained with FM4-64 in *wt* flies. (**B**) Binary image showing the ROIs segmented by gradient filtering of the boutons shown in A. (**C**) Histogram showing the areas of the objects detected in B. The black columns correspond to the area of the objects selected by size filtering. The object areas shown in the gray columns were not considered in the analyses.
(TIF)

Figure S3 **Activity-dependent exocytosis in the lamina.** (**A**) Boutons previously loaded with FM4-64 in the lamina are shown, before (**a**) and after (**b**) a second exposure to 90 mM K^+^. Fluorescence decay was detected in the boutons pointed out by arrowheads. (**Ac**) ROIs used for the quantification. (**B**) Mean fluorescence decay measured 3 min after exposure to 90 mM K^+^ in the ROIs shown in (Ac). Error bars: mean ± SEM.
(TIF)

Text S1 Detailed protocols for image capture and analysis.
(DOCX)

## References

[1] Hardie RC (1989) A histamine-activated chloride channel involved in neurotransmission at a photoreceptor synapse. Nature 339: 704–706. 2472552 10.1038/339704a0

[2] Stuart AE, Borycz J, Meinertzhagen IA (2007) The dynamics of signaling at the histaminergic photoreceptor synapse of arthropods. Prog Neurobiol 82: 202–227. 17531368 10.1016/j.pneurobio.2007.03.006

[3] Meinertzhagen IA, O’Neil SD (1991) Synaptic organization of columnar elements in the lamina of the wild type in *Drosophila melanogaster* . J Comp Neurol 305: 232–263. 1902848 10.1002/cne.903050206

[4] Hamanaka Y, Meinertzhagen IA (2010) Immunocytochemical localization of synaptic proteins to photoreceptor synapses of *Drosophila melanogaster* . J Comp Neurol 518: 1133–1155. 20127822 10.1002/cne.22268 PMC4029604

[5] Bloomquist BT, Shortridge RD, Schneuwly S, Perdew M, Montell C, et al (1988) Isolation of a putative phospholipase C gene of Drosophila, *norpA*, and its role in phototransduction. Cell 54: 723–733. 2457447 10.1016/s0092-8674(88)80017-5

[6] Hardie R, Postma M (2008) Phototransduction in microvillar photoreceptors of *Drosophila* and other invertebrates. In Basbaum AI, Kaneko A, Shepherd GM, Westheimer G, editors. The Senses: A comprehensive reference. SanDiego: Academic Press. 77–130.

[7] Niemeyer BA, Suzuki E, Scott K, Jalink K, Zuker CS (1996) The *Drosophila* light-activated conductance is composed of the two channels TRP and TRPL. Cell 85: 651–659. 8646774 10.1016/s0092-8674(00)81232-5

[8] Reuss H, Mojet MH, Chyb S, Hardie RC (1997) In vivo analysis of the *Drosophila* light-sensitive channels, TRP and TRPL. Neuron 19 1249–1259. 9427248 10.1016/s0896-6273(00)80416-x

[9] Chyb S, Raghu P, Hardie RC (1999) Polyunsaturated fatty acids activate the Drosophila light-sensitive channels TRP and TRPL. Nature 397: 255–259. 9930700 10.1038/16703

[10] Delgado R, Bacigalupo J (2009) Unitary recordings of TRP and TRPL channels from isolated Drosophila retinal photoreceptor rhabdomeres: activation by light and lipids. J Neurophysiol 101: 2372–2379. 19261713 10.1152/jn.90578.2008

[11] Lev S, Katz B, Tzarfaty V, Minke B (2012) Signal-dependent hydrolysis of phosphatidylinositol 4,5-biphosphate without activation of phospholipase C. Implications on gating of *Drosophila* TRPL (transient receptor potential-like) channel. JBC 287: 1436–1447. 10.1074/jbc.M111.266585 PMC3256851 22065576

[12] Estacion M, Sinkins WG, Schilling WP (2001) Regulation of *Drosophila* transient receptor potential-like (TRPL) channels by Phospholipase C-dependent mechanisms. J Physiol 530: 1–19. 11136854 10.1111/j.1469-7793.2001.0001m.x PMC2278390

[13] Huang J, Liu C-A, Hughes SA, Postma M, Schwiening CJ, et al (2010) Activation of TRP channels by protona and phosphoinositide depletion in *Dsosophila* photoreceptors. Curr Biol 20: 189–197. 20116246 10.1016/j.cub.2009.12.019

[14] Acharya JK, Jalink K, Hardy RW, Hartenstein V, Zuker CS (1997) InsP_3_ receptor is essential for growth and differentiation but not for vision in *Drosophila.* . Neuron 18: 881–887. 9208856 10.1016/s0896-6273(00)80328-1

[15] Raghu P, Colley NJ, Webel R, James T, Hasan G, et al (2000) Normal phototransduction in *Drosophila* photoreceptors lacking an InsP_3_ receptor gene. Mol Cell Neurosci 15: 429–445. 10833300 10.1006/mcne.2000.0846

[16] Xu XZ, Li HS, Guggino WB, Montell C (1997) Coassembly of TRP and TRPL produces a distinct store-operated conductance. Cell 89: 1155–1164. 9215637 10.1016/s0092-8674(00)80302-5

[17] Pollock JA, Assaf A, Peretz A, Nichols CD, Mojet MH, et al (1995) TRP, a protein essential for inositide-mediated Ca^2+^ influx is localized adjacent to the calcium stores in *Drosophila* photoreceptors. J Neurosci 15: 3747–3760. 7751943 10.1523/JNEUROSCI.15-05-03747.1995 PMC6578220

[18] Salido GM, Sage SO, Rosado JA (2009) Biochemical and functional properties of the store-operated Ca^2+^ channels. Cell Signal 21: 457–461. 19049864 10.1016/j.cellsig.2008.11.005

[19] Smith LA, Wang X, Peixoto AA, Neumann EK, Hall LM, et al (1996) A *Drosophila* calcium channel alpha1 subunit gene maps to a genetic locus associated with behavioral and visual defects. J Neurosci 16: 7868–7879. 8987815 10.1523/JNEUROSCI.16-24-07868.1996 PMC6579206

[20] Peng I-F, Wu C-F (2007) *Drosophila cacophony* Channels: A major mediator of neuronal Ca^2+^ currents and a trigger for K^+^ channels homeostatic regulation. J Neurosci 27: 1072–1081. 17267561 10.1523/JNEUROSCI.4746-06.2007 PMC6673189

[21] Moses K, Rubin GM (1991) Glass encodes a site-specific DNA-binding protein that is regulated in response to positional signals in the developing Drosophila eye. Genes Dev 5: 583–593. 2010085 10.1101/gad.5.4.583

[22] Ramírez O, Vidal R, Tello J, Vargas K, Kindler S, et al (2009) Dendritic assembly of heteromeric Gaba_b_ receptor subunits in hippocampal neurons. J Biol Chem 284: 13077–13085. 19276079 10.1074/jbc.M900575200 PMC2676040

[23] Ramírez O, Rojas R, Couve A, Härtel S (2010) Confined displacement algorithm determines true and random colocalization in fluorescence microscopy. J Microsc 239: 173–183. 20701655 10.1111/j.1365-2818.2010.03369.x

[24] Wang Y, Guo HF, Pologruto TA, Hannan F, Hakker I, et al (2004) Stereotyped odor-evoked activity in the mushroom body of *Drosophila* revealed by green fluorescent protein-based Ca^2+^ imaging. J Neurosci 24: 6507–6514. 15269261 10.1523/JNEUROSCI.3727-03.2004 PMC6729867

[25] Leung HT, Branton WD, Phillips HS, Jan L, Byerly L (1989) Spider toxins selectively block calcium currents in *Drosophila.* . Neuron 3: 767–772. 2642017 10.1016/0896-6273(89)90245-6

[26] King GF (2007) Modulation of insect Ca(v) channels by peptidic spider toxins. Toxicon 49: 513–530. 17197008 10.1016/j.toxicon.2006.11.012

[27] Peixoto AA, Hall JC (1998) Analysis of temperature-sensitive mutants reveals new genes involved in the courtship song of *Drosophila.* . Genetics 148: 827–838. 9504928 10.1093/genetics/148.2.827 PMC1459814

[28] Running Deer JL, Hurley JB, Yarfitz SL (1995) G protein control of *Drosophila* photoreceptor phospholipase C. J Biol Chem. 270: 12623–12628. 10.1074/jbc.270.21.12623 7759511

[29] Bird GS, Wayne I DeHaven, Jeremy T Smyth, et al (2008) Methods for studying store-operated calcium entry. Methods 46: 204–212. 18929662 10.1016/j.ymeth.2008.09.009 PMC2643845

[30] Gu H, Jiang SA (2009) Campusano JM, Iniguez J, Su H, et al (2009) Cav2-type calcium channels encoded by *cac* regulate AP-independent neurotransmitter release at cholinergic synapses in adult *Drosophila* brain. J Neurophysiol 101: 42–53. 19004991 10.1152/jn.91103.2008 PMC2637009

[31] Dickman DK, Kurshan PT, Schwarz TL (2008) Mutations in a *Drosophila* alpha2delta voltage-gated calcium channel subunit reveal a crucial synaptic function. J Neurosci 28: 31–38. 18171920 10.1523/JNEUROSCI.4498-07.2008 PMC6671140

[32] Smith LA, Peixoto AA, Hall JC (1998) RNA editing in the *Drosophila* DMCA1A calcium-channel alpha-1 subunit transcript. J Neurogenetics 12: 227–240. 10656110 10.3109/01677069809108560

[33] Pyza E (2002) Dynamic structural changes of synaptic contacts in the visual system of insects. Microsc Res Tech 58: 335–344. 12214300 10.1002/jemt.10141

[34] Hardie RC, Gu Y, Martin F, Sweeney ST, Raghu P (2004) In vivo light-induced and basal phospholipase C activity in *Drosophila* photoreceptors measured with genetically targeted phosphatidylinositol 4,5-bisphosphate-sensitive ion channels (Kir2.1). J Biol Chem 279: 47773–47782. 15355960 10.1074/jbc.M407525200

[35] Ryglewski S, Pflueger HJ, Duch K (2007) Expanding the neuronśs calcium signalling repertoire: intracellular calcium release via voltage-induced PLC and IP_3_R activation. PloS Biol 5: 818–827. 10.1371/journal.pbio.0050066 PMC1808487 17341135

[36] Gu Y, Oberwinkler J, Postma M, Hardie RC (2005) Mechanisms of light adaptation in *Drosophila* photoreceptors. Curr Biol 15: 1228–1234. 16005297 10.1016/j.cub.2005.05.058

[37] Venkatachalam K, Montell C (2007) TRP channels. Annu Rev Biochem 76: 387–417. 17579562 10.1146/annurev.biochem.75.103004.142819 PMC4196875

[38] Szikra T, Cusato K, Thoreson WB, Barabas P, Bartoletti TM, et al (2008) Depletion of calcium stores regulates calcium influx and signal transmission in rod photoreceptors. J Physiol 586: 4859–4875. 18755743 10.1113/jphysiol.2008.160051 PMC2614069

[39] Agrawal N, Venkiteswaran G, Sadaf S, Padmanabhan N, Banerjee S, et al (2010) Inositol 1,4,5-trisphosphate receptor and dSTIM function in *Drosophila* insulin-producing neurons regulates systemic intracellular calcium homeostasis and flight. J Neurosci 30: 1301–1313. 20107057 10.1523/JNEUROSCI.3668-09.2010 PMC6633787

[40] Yuan JP, Zeng W, Huang GN, Worley PF, Muallem S (2007) STIM1 heteromultimerizes TRPC channels to determine their function as store-operated channels. Nat Cell Biol 9: 636–645. 17486119 10.1038/ncb1590 PMC2699187

[41] LoGiudice L, Matthews G (2009) The role of ribbons at sensory synapses. Neuroscientist 15: 380–391. 19264728 10.1177/1073858408331373 PMC2743156

[42] Vázquez-Martínez O, Canedo-Merino R, Díaz-Munoz M, Riesgo-Escovar JR (2003) Biochemical characterization, distribution and phylogenetic analysis of *Drosophila melanogaster* ryanodine and IP_3_ receptors, and thapsigargin-sensitive Ca^2+^ ATPase. J Cell Sci 116: 2483–2494. 12766186 10.1242/jcs.00455

[43] Cadetti L, Bryson EJ, Ciccone CA, Rabl K, Thoreson WB (2006) Calcium-induced calcium release in rod photoreceptor terminals boosts synaptic transmission during maintained depolarization. Eur J Neurosci 23: 2983–2990. 16819987 10.1111/j.1460-9568.2006.04845.x PMC2474468

[44] Weckström M, Juusola M, Uusitalo RO, French AS (1995) Fast-acting compressive and facilitatory nonlinearities in light-adapted fly photoreceptors. Ann Biomed Eng 23: 70–77. 7762884 10.1007/BF02368302

[45] Wang T, Xu H, Oberwinkler J, Gu Y, Hardie RC, et al (2005) Light activation, adaptation, and cell survival functions of the Na^+^/Ca^2+^ exchanger CaIX. Neuron 45: 367–378. 15694324 10.1016/j.neuron.2004.12.046

[46] Franceschini N, Kirschfeld K (1971) Pseudopupil phenomena in the compound eye of *Drosophila.* . Kybernetik 9: 159–182. 5134358 10.1007/BF02215177

[47] Ortiz-Miranda SI, Dayanithi R, Velazquez-Marrero C, Custer EE, Treistman SN, et al (2010) Differential Modulation of N-Type Calcium Channels by micro-Opioid Receptors in Oxytocinergic Versus Vasopressinergic Neurohypophysial Terminals. J Cell Physiol 225: 276–288. 20509142 10.1002/jcp.22263 PMC4060829

[48] Smith DP, Ranganathan R, Hardy RW, Marx J, Tsuchida T, et al (1991) Photoreceptor deactivation and retinal degeneration mediated by a photoreceptor-specific protein kinase C. Science. 254: 1478–1484. 10.1126/science.1962207 1962207

[49] Tsunoda S, Sierralta J, Sun Y, Bodner R, Suzuki E, et al (1997) A multivalent PDZ-domain protein assembles signalling complexes in a G-protein-coupled cascade. Nature 388: 243–249. 9230432 10.1038/40805

[50] Delgado R, Maureira C, Oliva C, Kidokoro Y, Labarca P (2000) Size of vesicle pools, rates of mobilization, and recycling at neuromuscular synapses of a *Drosophila* mutant, *shibire.* . Neuron 28: 941–953. 11163278 10.1016/s0896-6273(00)00165-3

