# Supplementary figures and images for: TRP, TRPL and Cacophony Channels Mediate Ca2+ Influx and Exocytosis in Photoreceptors Axons in Drosophila
Source: PLoS One. 2012 Aug 31;7(8):e44182. doi: 10.1371/journal.pone.0044182 (PMC3432082; doi:10.1371/journal.pone.0044182)

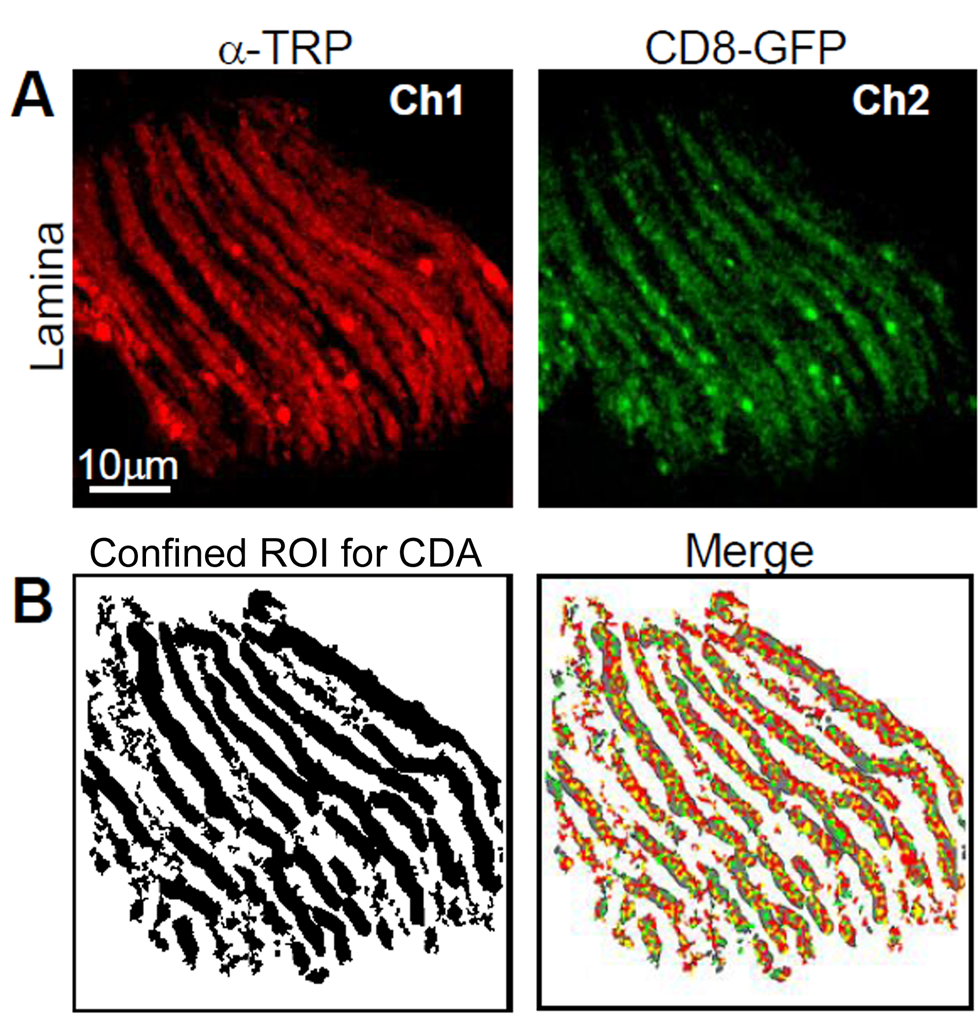

Supplement: Figure S1 — Segmentation of ROIs for colocalization analysis by the confined displacement algorithm (CDA). (A) Representative 2-channel confocal image with α-TRP (Ch1, red, left) and mCD8-GFP (Ch 2, green, right). (B) Confined region defines the photoreceptors axons (from Ch2) after segmentation (left). Merge of the segmented signals for Ch1 (red) and Ch2 (green) within the confined ROI (grey) (right). (TIF) [file pone.0044182.s001.tif]

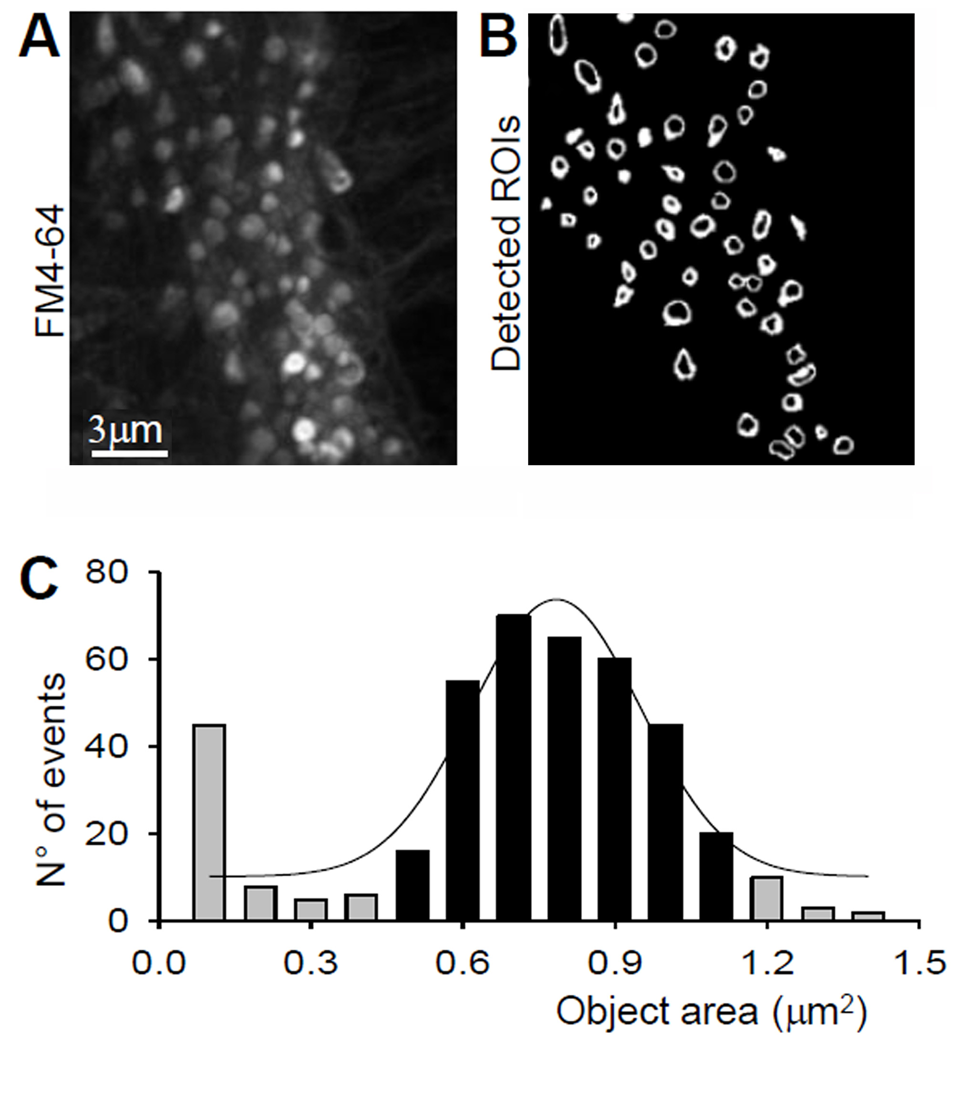

Supplement: Figure S2 — Bouton detection and quantification in confocal images. (A) Representative image of lamina boutons stained with FM4-64 in wt flies. (B) Binary image showing the ROIs segmented by gradient filtering of the boutons shown in A. (C) Histogram showing the areas of the objects detected in B. The black columns correspond to the area of the objects selected by size filtering. The object areas shown in the gray columns were not considered in the analyses. (TIF) [file pone.0044182.s002.tif]

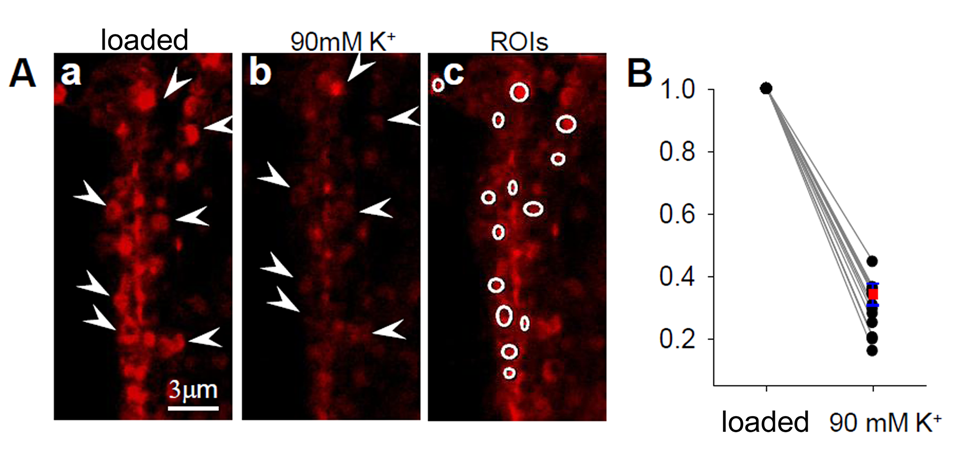

Supplement: Figure S3 — Activity-dependent exocytosis in the lamina. (A) Boutons previously loaded with FM4-64 in the lamina are shown, before (a) and after (b) a second exposure to 90 mM K+. Fluorescence decay was detected in the boutons pointed out by arrowheads. (Ac) ROIs used for the quantification. (B) Mean fluorescence decay measured 3 min after exposure to 90 mM K+ in the ROIs shown in (Ac). Error bars: mean ± SEM. (TIF) [file pone.0044182.s003.tif]
