# Supplementary material for: TRP, TRPL and Cacophony Channels Mediate Ca2+ Influx and Exocytosis in Photoreceptors Axons in Drosophila
Source: PLoS One. 2012 Aug 31;7(8):e44182. doi: 10.1371/journal.pone.0044182 (PMC3432082; doi:10.1371/journal.pone.0044182)
Supplement: Text S1 — Detailed protocols for image capture and analysis. (DOCX) [file pone.0044182.s004.docx]

**Supporting Text ST**

**Image capture and analysis.**

Images were acquired in 8 bits with a Zeiss LSM Meta 510 confocal microscope using LSM 5 3.2 image capture and analysis software (Zeiss). Objectives 20x/0.5 NA, 40x/0.75 NA, 63x/1.4 NA oil digital zoom (4x) and 100x/1.3 NA oil digital zoom (3x) were used.

Ca2+ images were acquired at 8 bits in one channel (excitation/emission wavelength λ_exc_/λ_em_ = 488/505-530 nm for G-CaMP and 543/560-590 nm for Rhod-2). For quantification, 10 z-stack confocal images (in x/y axis, 36 x 36 µm^2^ size) were acquired before, during and after treatments in the lamina or the complete visual system. Images were deconvolved as described below and z-projections were used for subsequent analysis using ImageJ software (National Institutes of Health). Fluorescence measurements in the resulting images were performed using the "Integrated Density" option which corresponds to the mean fluorescence value in the region of interest (ROI) multiplied by the ROI area. Data are expressed as ΔF/F_0_.

For FM4-64 experiments ten confocal z-stack images were acquired at Δz = 0.3 µm steps over a 36.56 µm sided square ROI in one channel (λ_exc_/λ_em_ = 543/590-615 nm). These images were projected in z for analysis and bouton quantification (see below). For immunofluorescence experiments, two channel- image stacks were acquired in the multi-track mode (intensity I(x,y,z) ∈[0,255], voxel size Δx/Δy/Δz = 70/70/300 nm, Channel-1 (Ch1) λ_exc_/λ_em_ = 488/505-530 nm, and channel-2 (Ch2) λ_exc_/λ_em_ = 543/560-590 or 543/590-615 nm. I(x,y,z) did not saturate and image background was set slightly above zero by adjusting the laser power, detector gain and offset.

Raw confocal image stacks were deconvolved by Huygens Scripting software (Scientific Volume Imaging, Hilversum, Netherlands) using an algorithm based on the Classic Maximum Likelihood Estimator. Image-processing routines were developed in SCIAN laboratory (www.scian.cl) based on interactive data language (IDL, ITT, Boulder, Colorado). These procedures were used for ROI segmentation, quantification, and to determine colocalization coefficients (see below).

**Selection of FM4-64 labeled synaptic boutons for quantification.**

Deconvolved projections of 10 z-stack images (Δz = 0.3μm) were segmented to select binary ROIs by application of two different filters:

1) *Gradient filter,* to select high S/N objects from background*.* This procedure was used to determine object contours and to separate contiguous regions within a 10 pixel radius.

2) *Size selection filter* to choose objects between 0.5 to 1.1 µm^2^, the most abundant bouton population as determined by staining with FM4-64 (Fig. S1).

**Analysis of colocalization of immunofluorescence signals.**

ROIs for colocalization analyses of immunofluorescence images of the lamina were determined by processing deconvolved images by gradient filters (Laplace) and thresholds were selected within the histograms according to Ramirez et al. [22,23]. As reported, Laplace filter enhance object contours within a heterogeneous background scenario and lead to a robust segmentation of fluorescent point sources, cellular organelles, or tubular structures like dendrites or axons. In addition, Cartridges, axons and boutons [3] were consistently selected with this procedure in the lamina. The segmentation procedures were checked vigorously by overlaying the original images with the respective ROIs and applied to all images within an experiment.

For colocalization analysis, segmented cartridges confined the displacement area, and the confined displacement algorithm (CDA) was performed according to [23]. Manders coefficients M1 (Eq. 1) were calculated within defined ROIs (M1_ROI_) and the ratio of colocalization to total signal was determined in a channel-specific manner.

Eq. 1

CDA introduces radial displacements in the xy-plane, leading to random scenarios in each channel without comprising the spatial correlation of fluorescence signal patterns. This 2D image correlation technique calculates M1_ROI_ as a function of radial displacements d, M1_ROI_(d):

$$M1_{ROI}\left(d\right)=M1_{ROI}\left(x',y'|\sqrt{x'^{2}+y'^{2}}=d\right)=\frac{\sum_{ij} I_{Ch1(ROI1\left(xi,yj\right)\cap ROI2\left(xi+x',yj+y'\right))}}{\sum_{ij} I_{Ch1(ROI1\left(xi,yj\right))}}$$

Eq. 2

We restricted the colocalization analysis to the xy-plane since the confocal point spread function (PSF) presents an elongation factor of about three in the z-axis, which lowers the precision for resolution and hence colocalization by the same factor. For the xy-plane the precision of the CDA is limited by the diffraction of the imaging system (about half width of the PSF). For a maximum displacement d’, pixels are shifted within: $\sqrt{x'^{2}+y'^{2}}\leq d':x'\in[-d',d'],y'\in[-d',d']$. For each shift, one data value is obtained as a function of the Euclidian distance$d=\sqrt{x'^{2}+y'^{2}}$. Successive shifts lead to one value for d = 0, four values for d = 1, d =$\sqrt{2}$ and d = 2, eight values for d =$\sqrt{5}$, etc. With increasing displacement, M1_ROI_(d) asymptotically approaches random scenarios when d exceeds the size of the colocalizing ROIs M1_ROI_(random). From the random values, probability density functions can be calculated in order to test whether the original M1_ROI_ (d = 0) is statistically different from a random population in terms of *p*-values. In addition, we can calculate the effective colocalization M1_ROI_(effective) = M1_ROI_(d = 0) - M1_ROI_(random) in order to correct the coefficients for the random colocalization which fluctuates with the signal density within the confined regions.
